# Supplementary figures and images for: CDCP1 promotes compensatory renal growth by integrating Src and Met signaling
Source: Life Sci Alliance. 2021 Feb 11;4(4):e202000832. doi: 10.26508/lsa.202000832 (PMC7893822; doi:10.26508/lsa.202000832)

Figure S1D

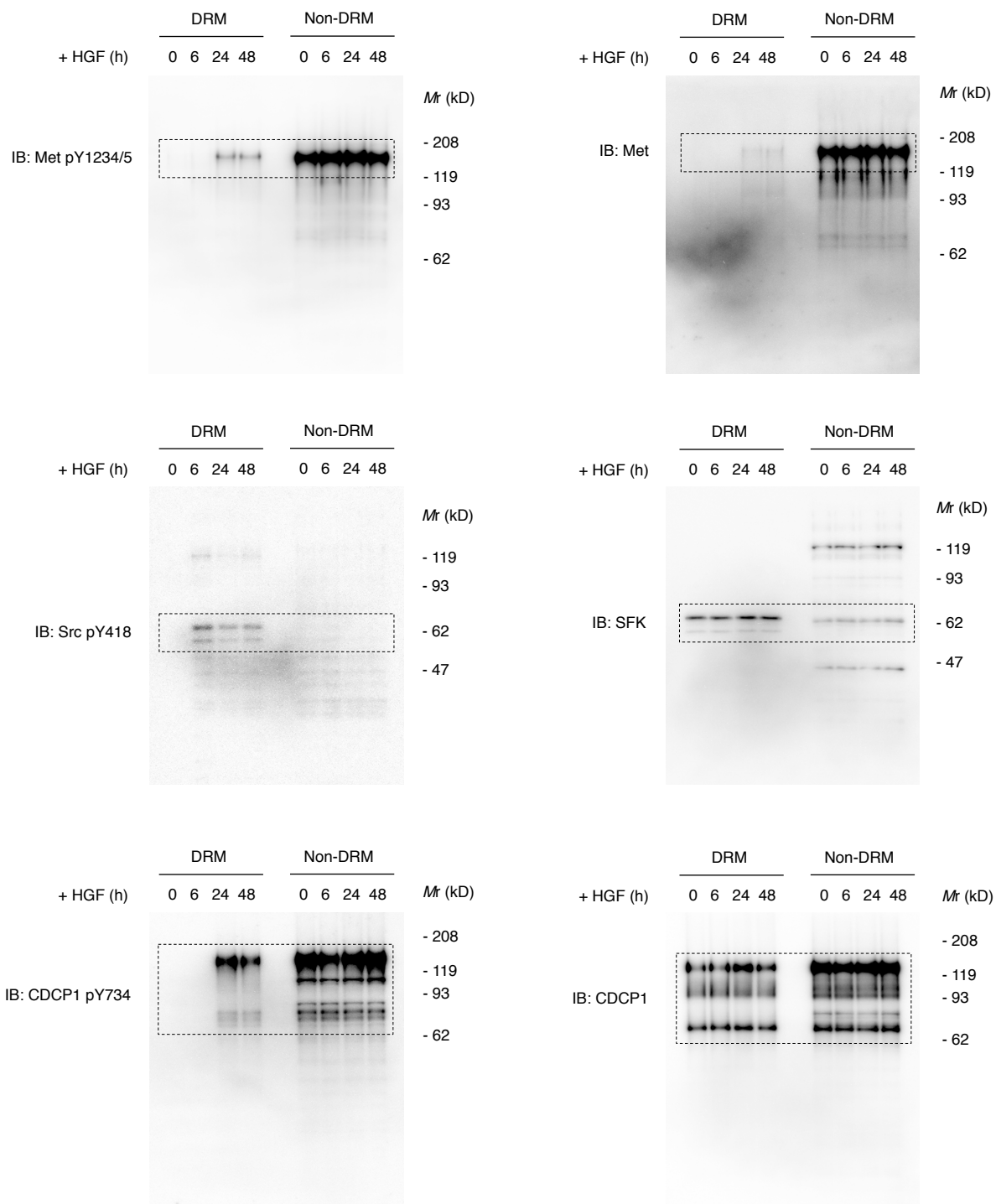

Supplement: Supplementary file 1 [file LSA-2020-00832_SdataFS1.pdf]

Figure S2B

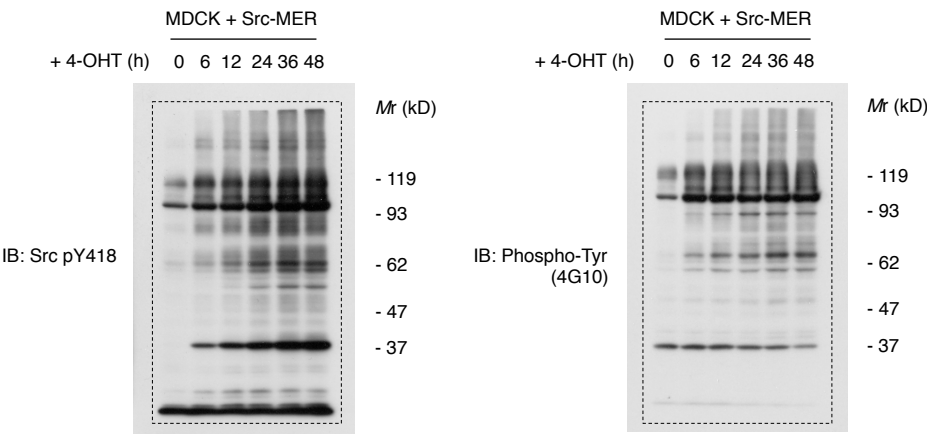

Figure S2D

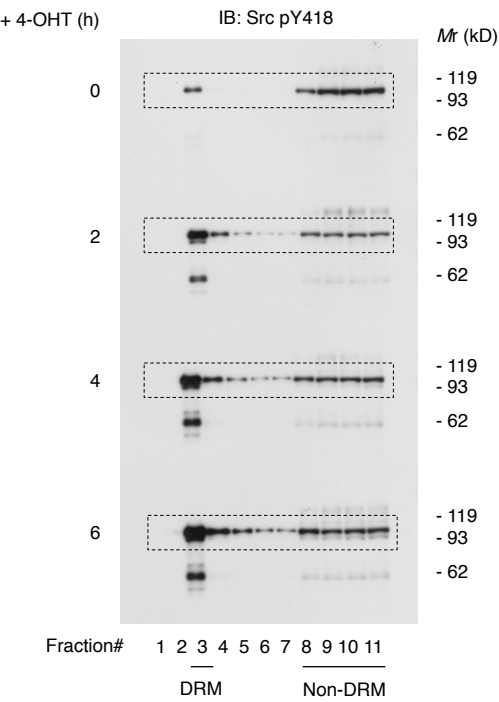

Supplement: Supplementary file 2 [file LSA-2020-00832_SdataFS2.pdf]

Figure S3C

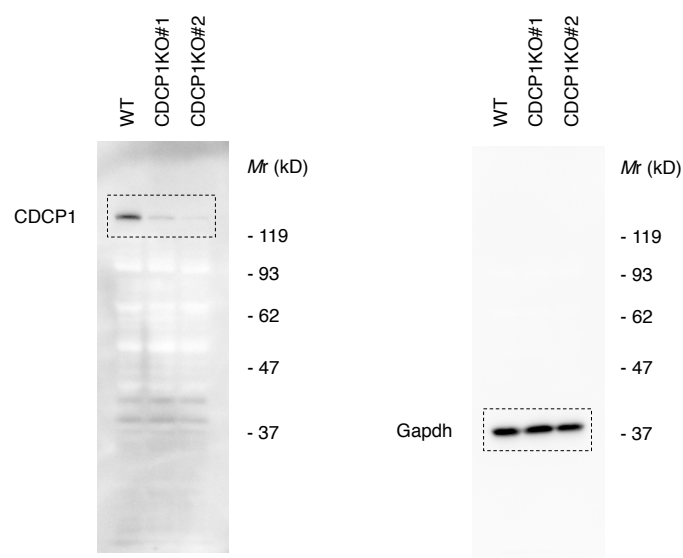

Supplement: Supplementary file 3 [file LSA-2020-00832_SdataFS3.pdf]

Figure S4A

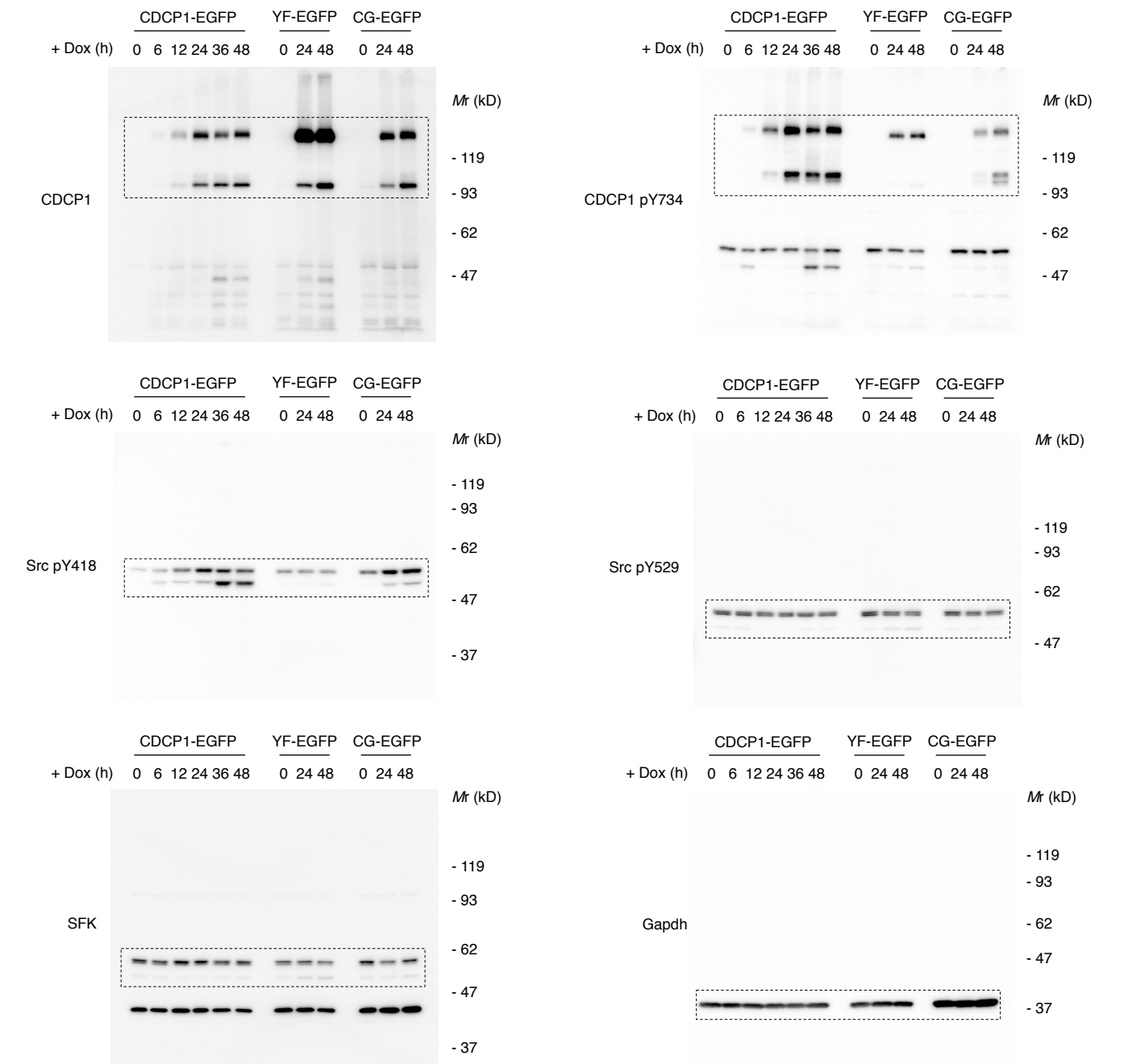

Figure S4C

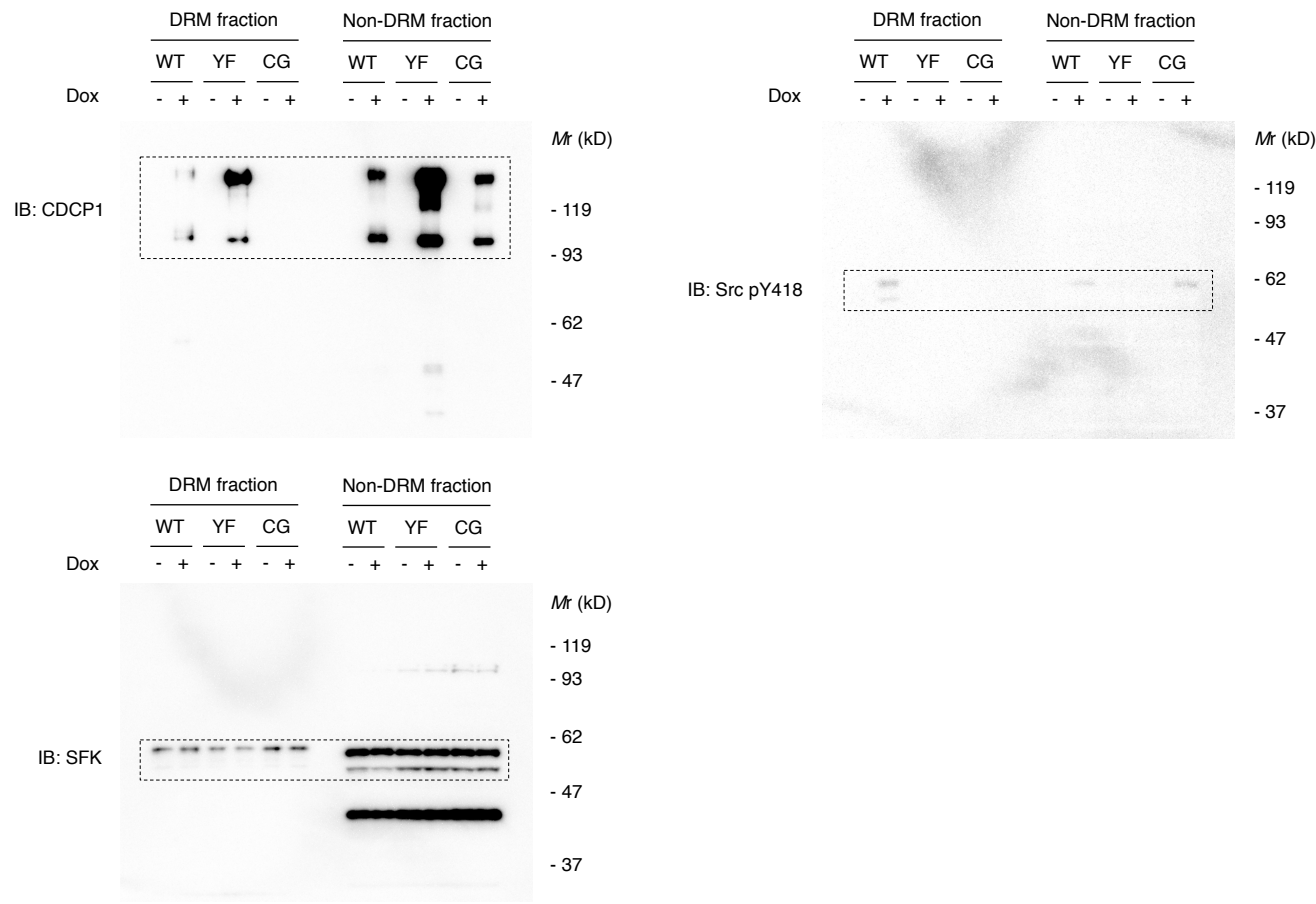

Figure S4D

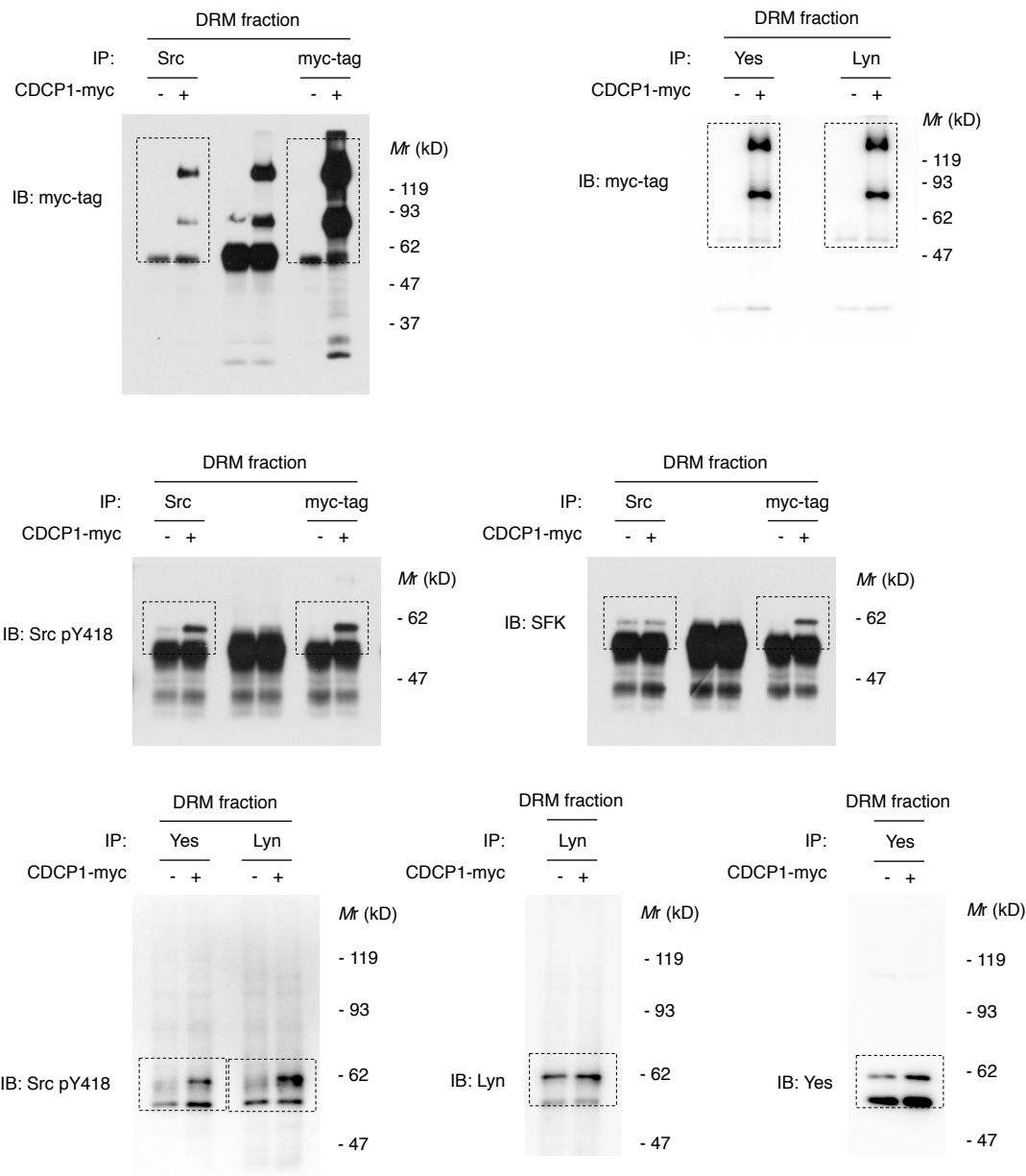

Figure S4F

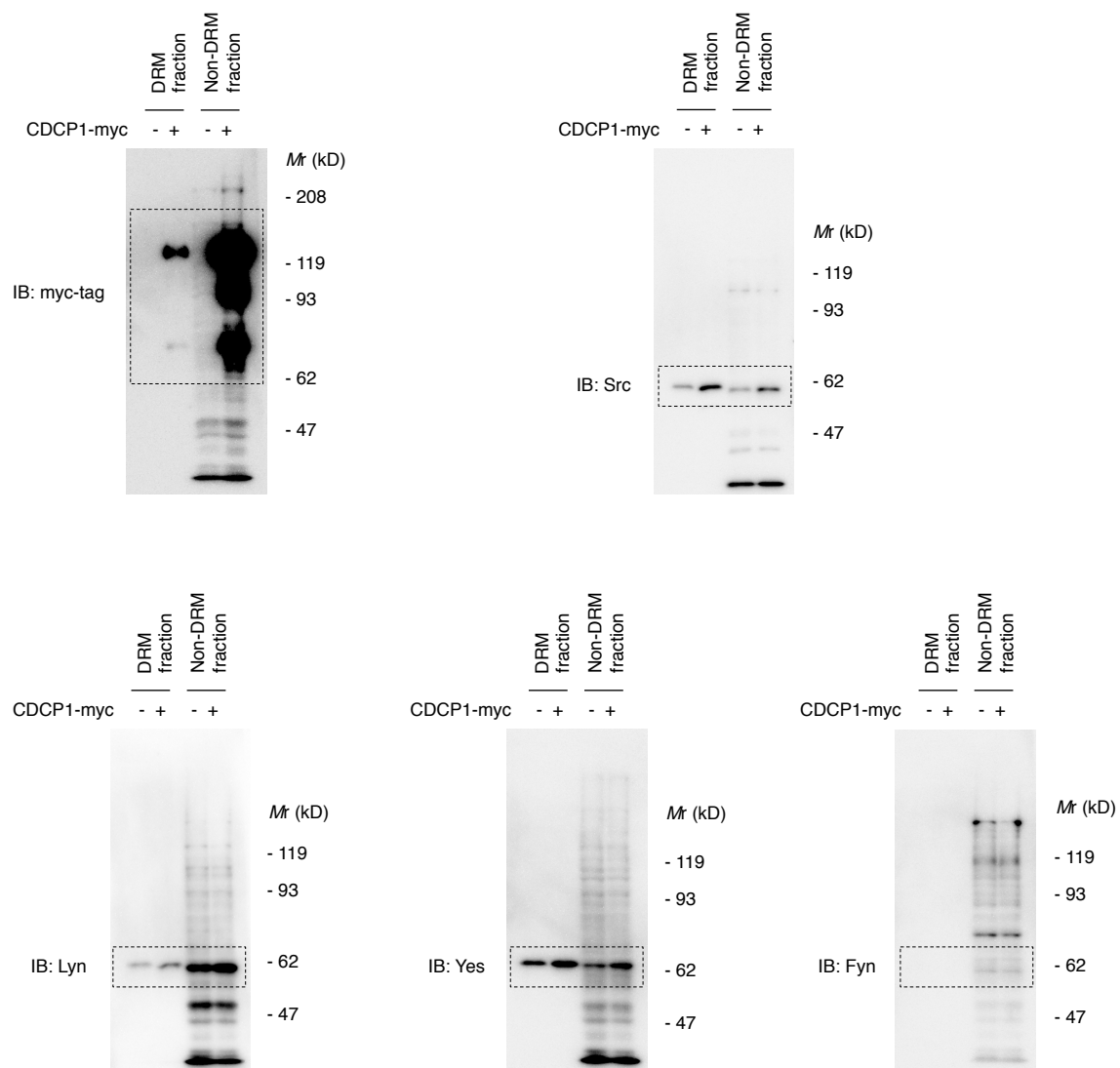

Supplement: Supplementary file 4 [file LSA-2020-00832_SdataFS4.pdf]

Figure 3A

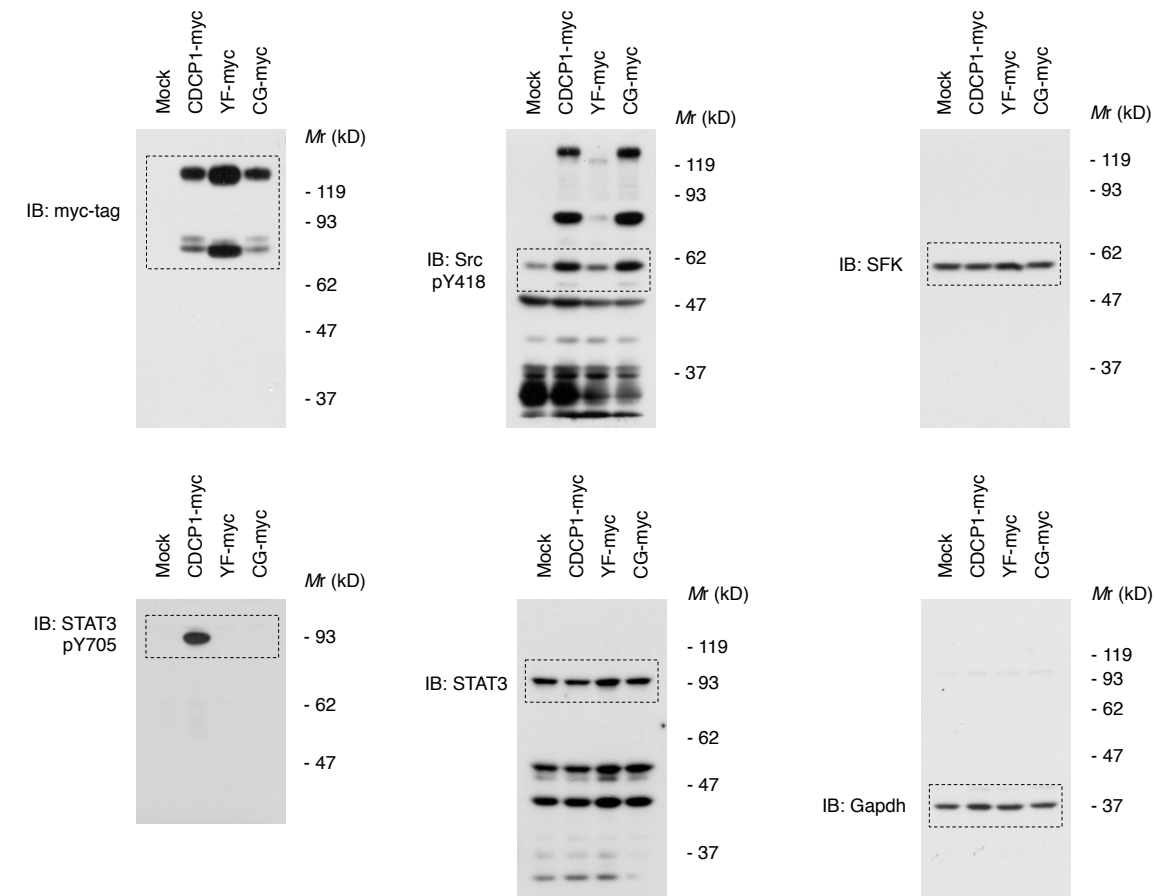

Supplement: Supplementary file 9 [file LSA-2020-00832_SdataF3.pdf]

Figure S7B

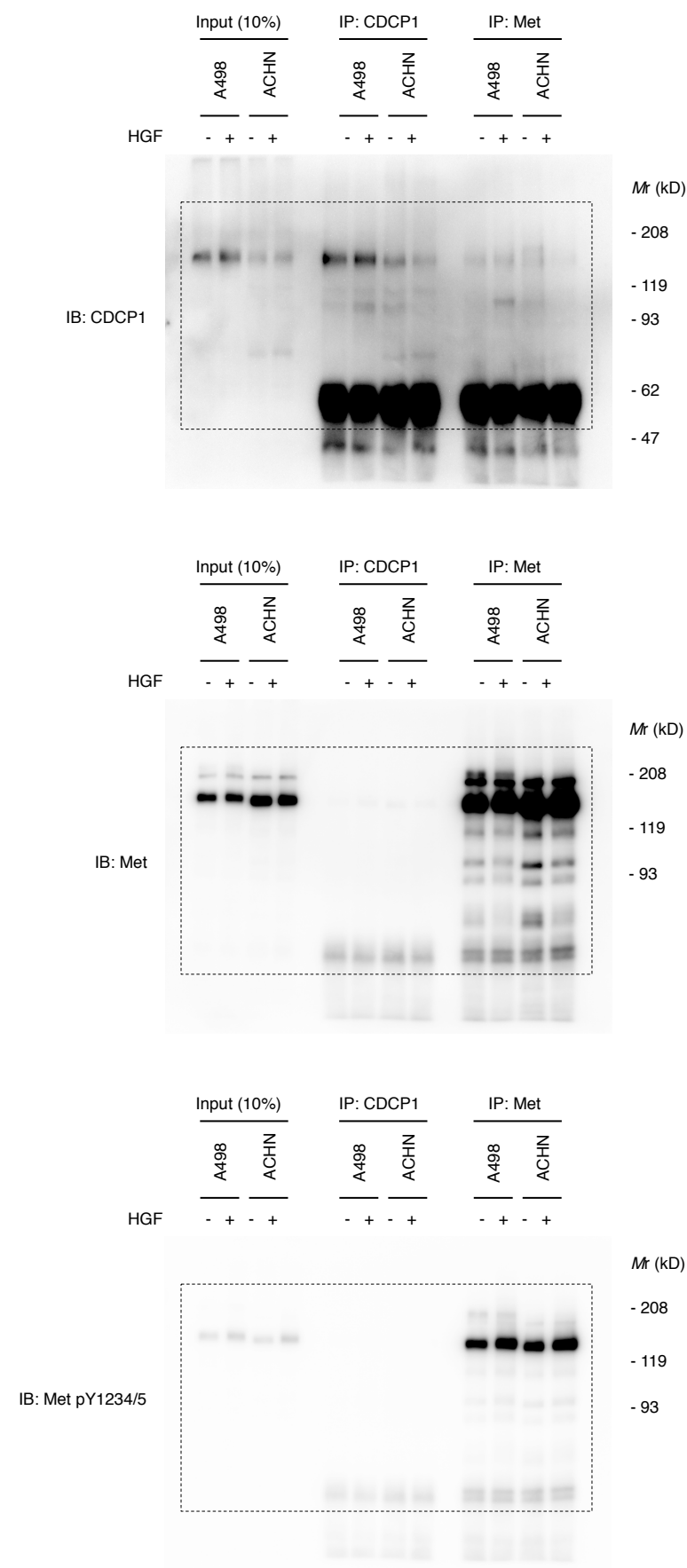

Supplement: Supplementary file 10 [file LSA-2020-00832_SdataFS7.pdf]

Figure 4C

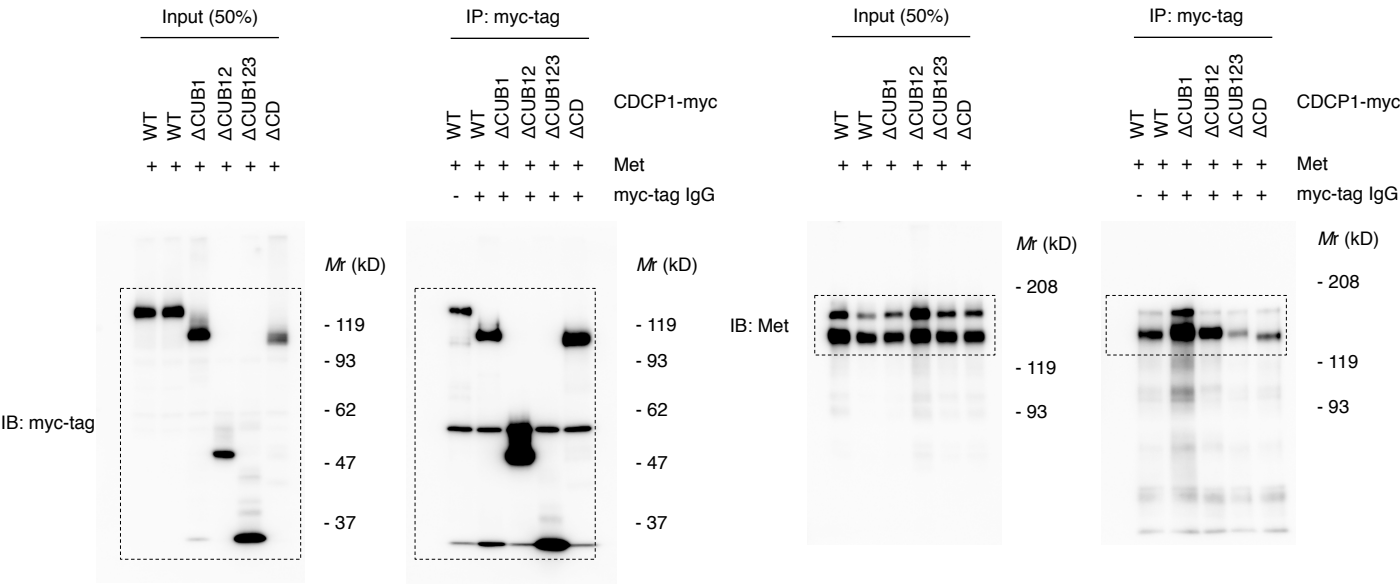

Figure 4D

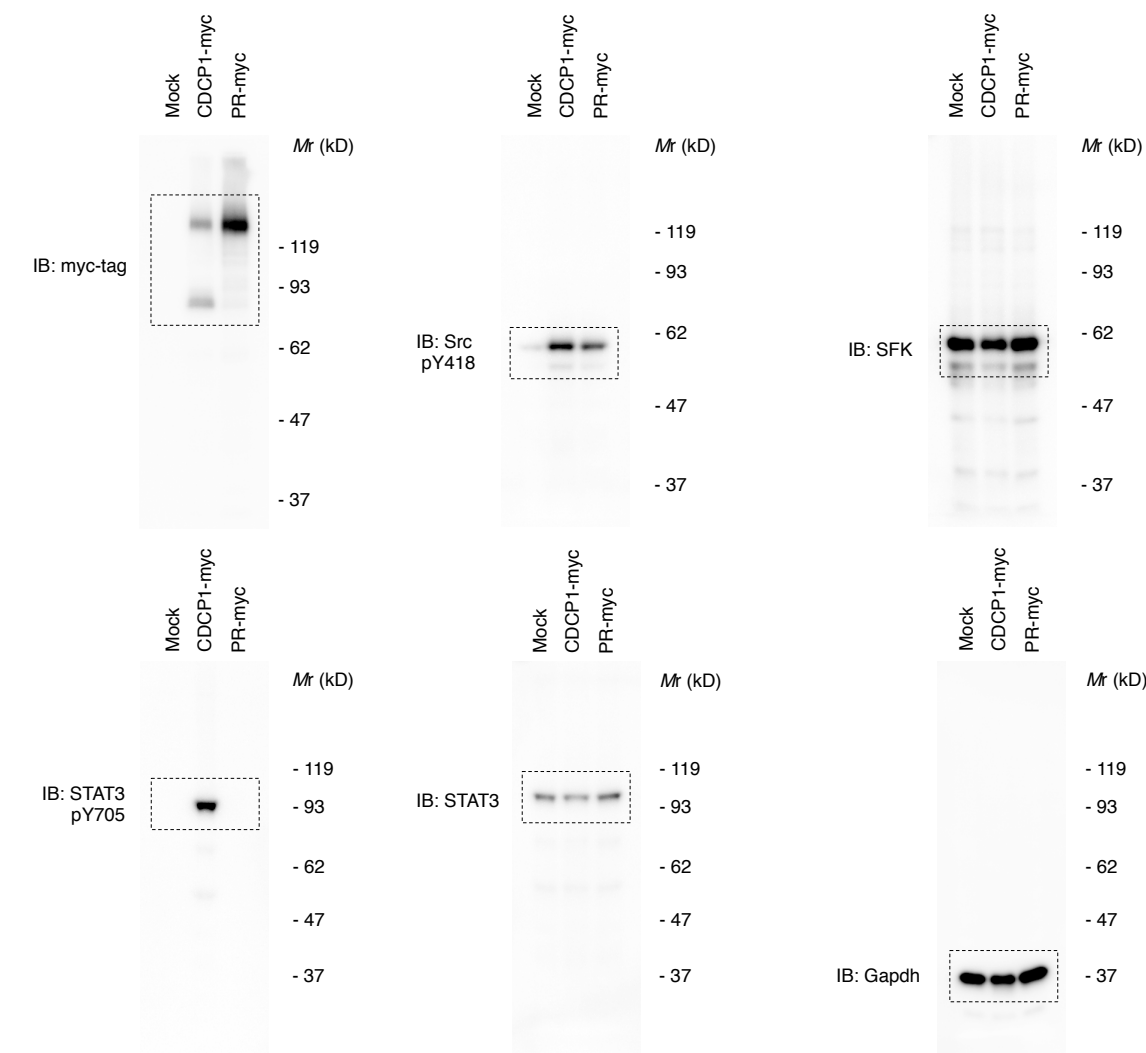

Supplement: Supplementary file 11 [file LSA-2020-00832_SdataF4.pdf]

Figure 5A

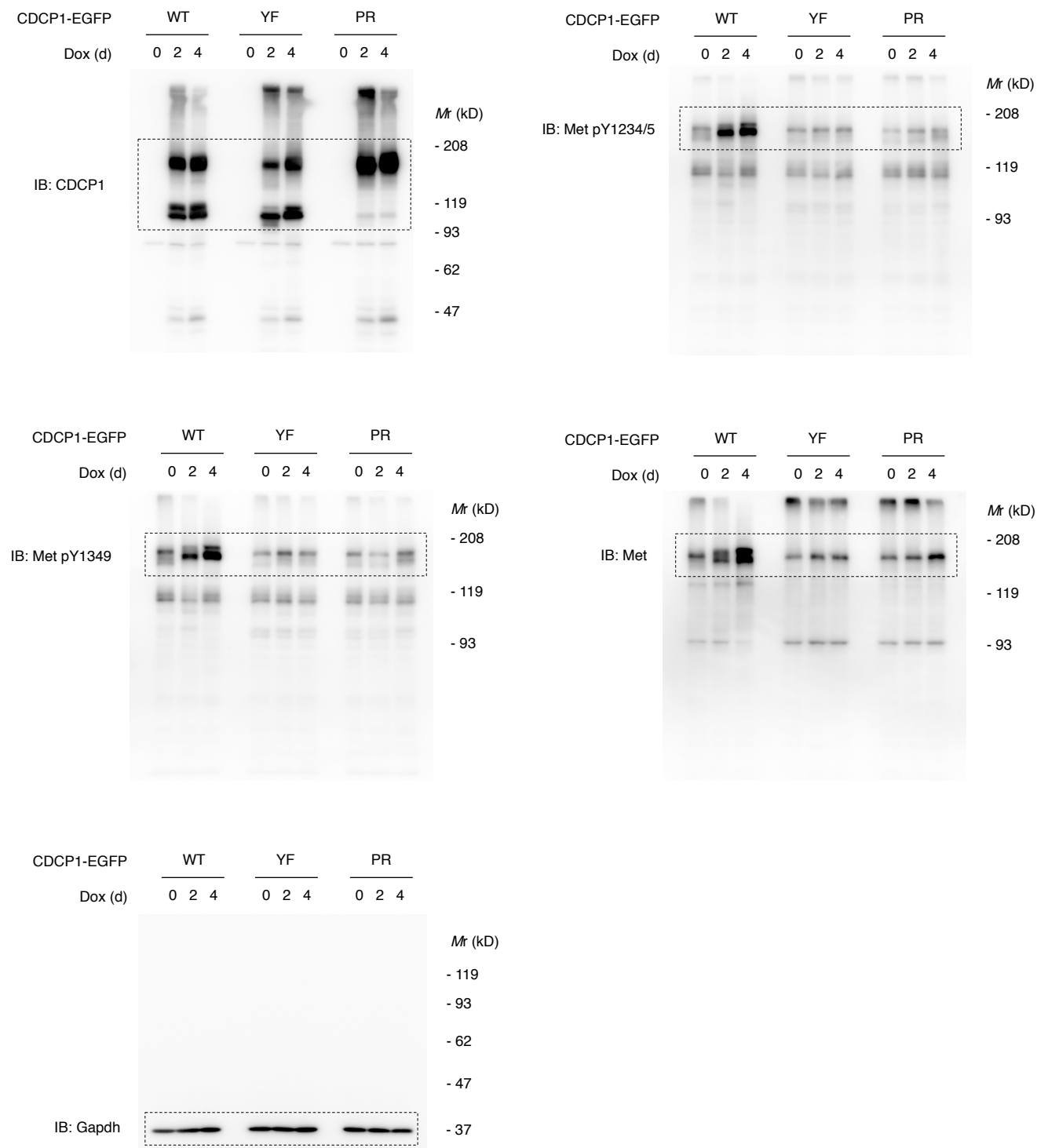

Figure 5E

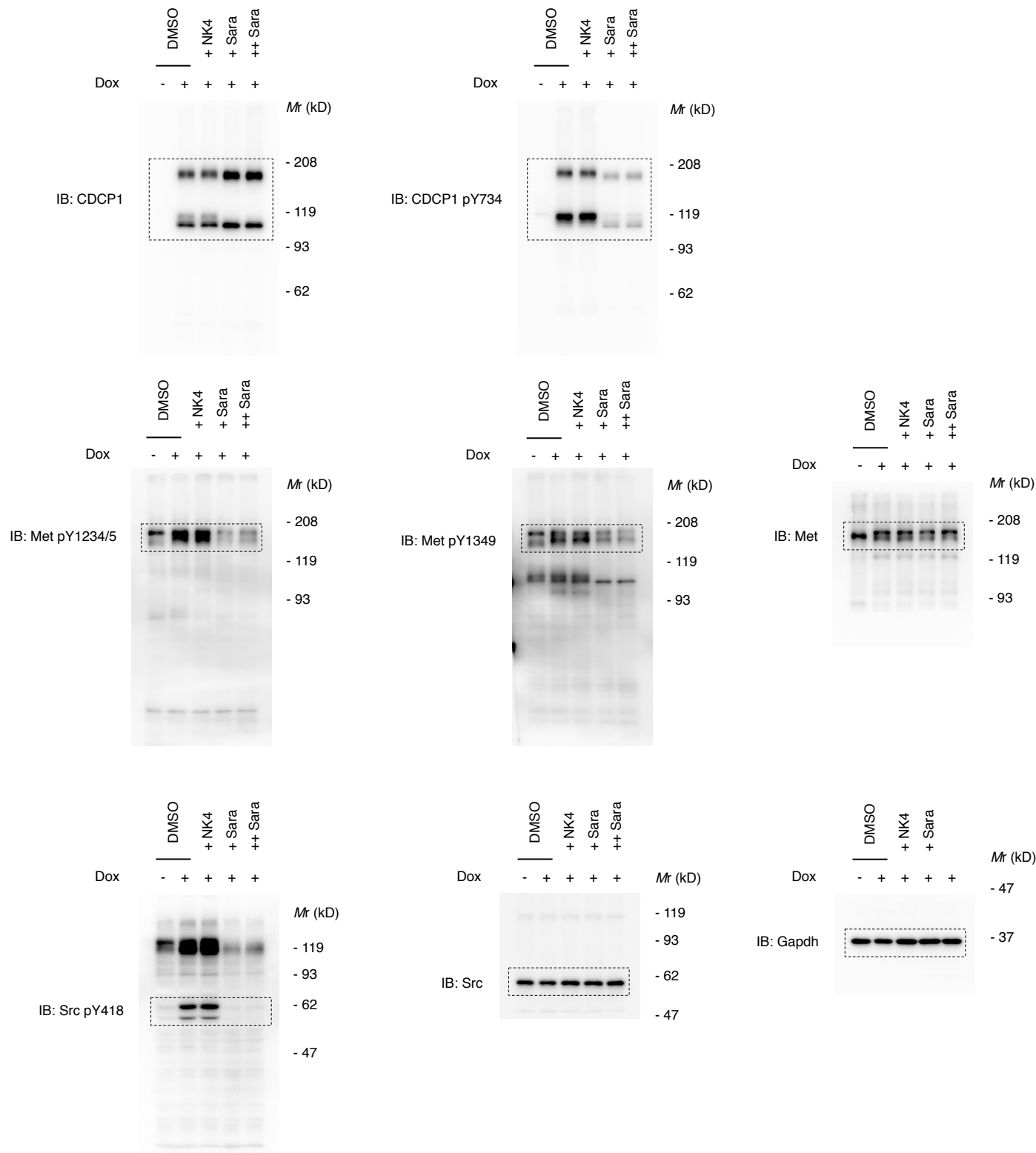

Figure 5G

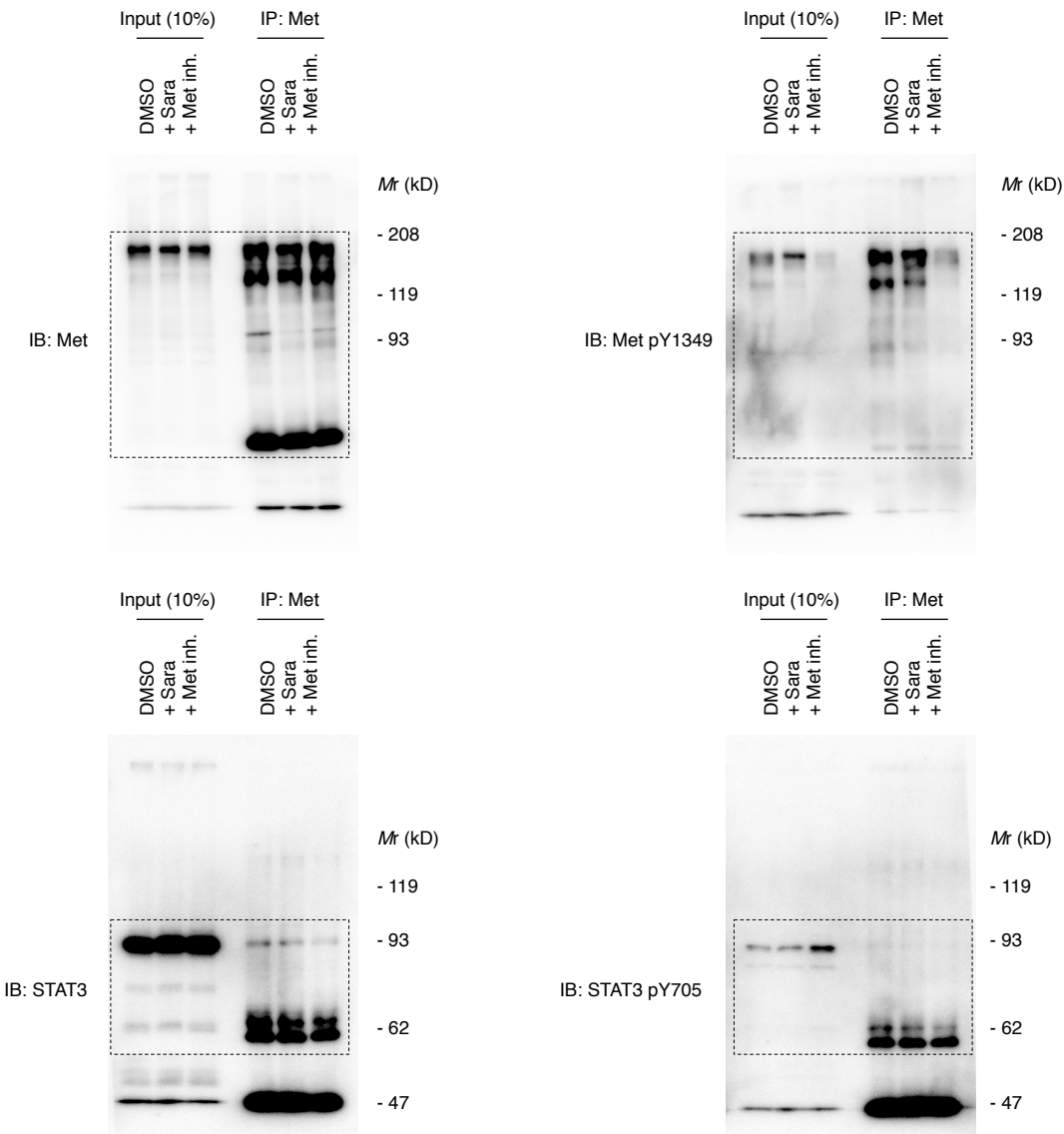

Supplement: Supplementary file 12 [file LSA-2020-00832_SdataF5.pdf]

Figure S8A

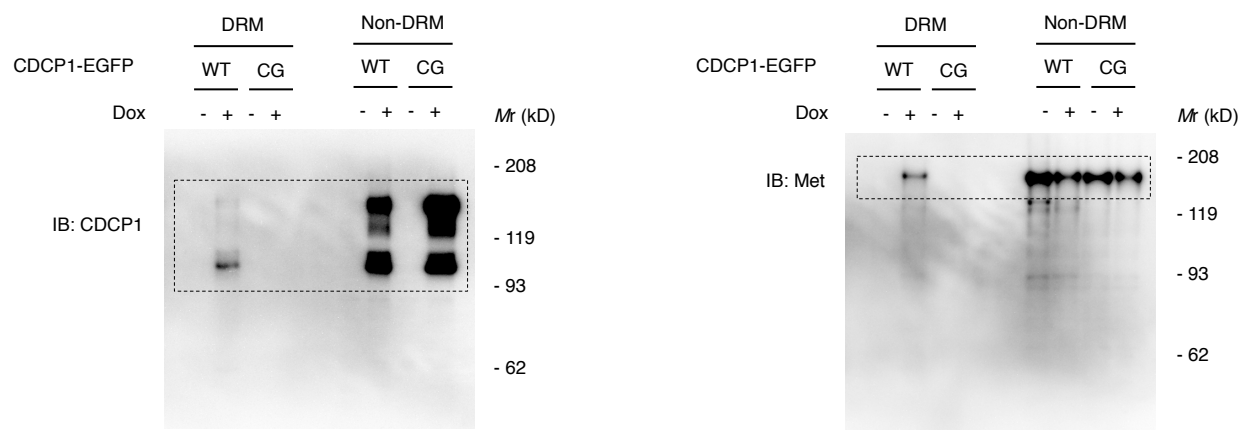

Figure S8G

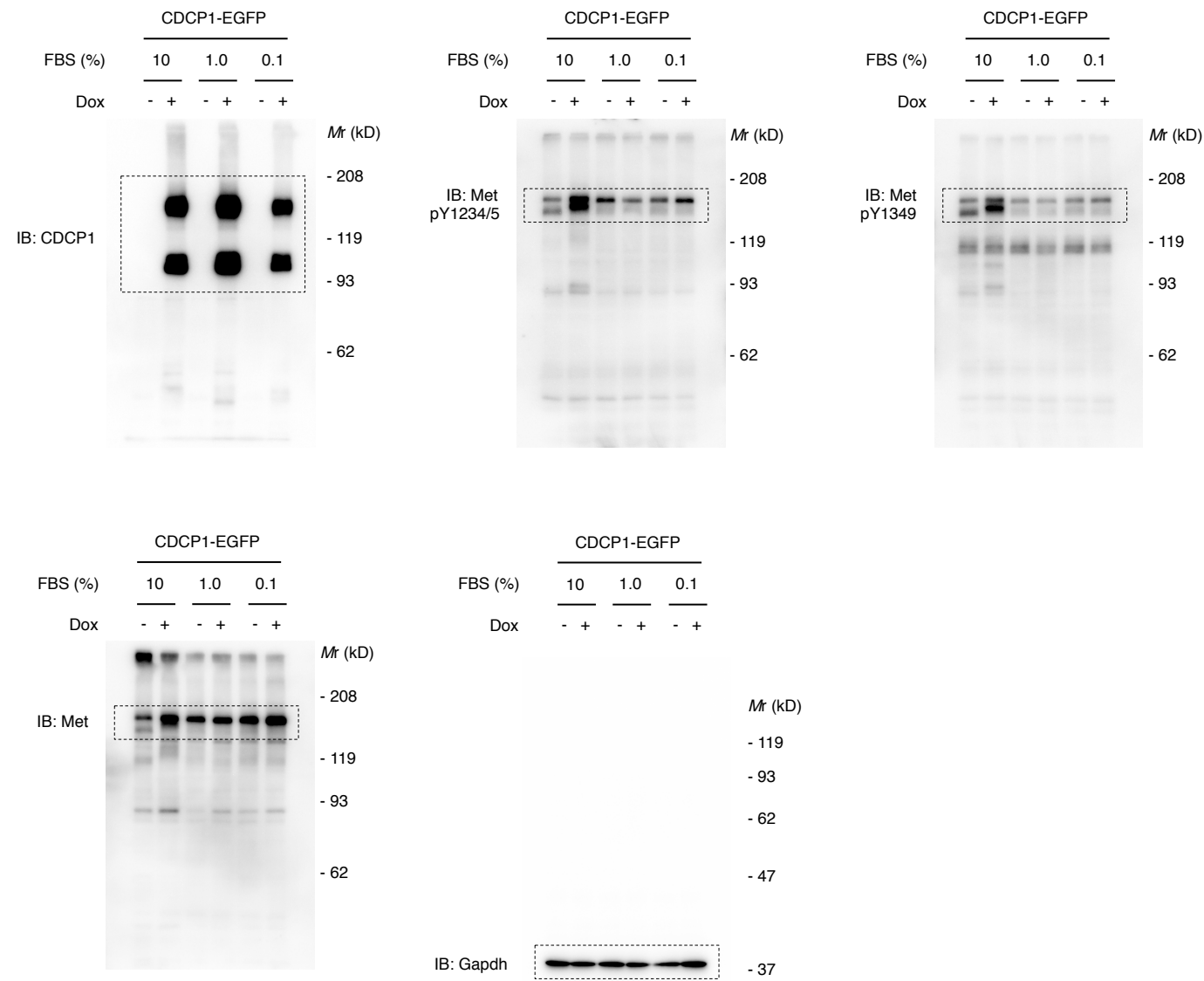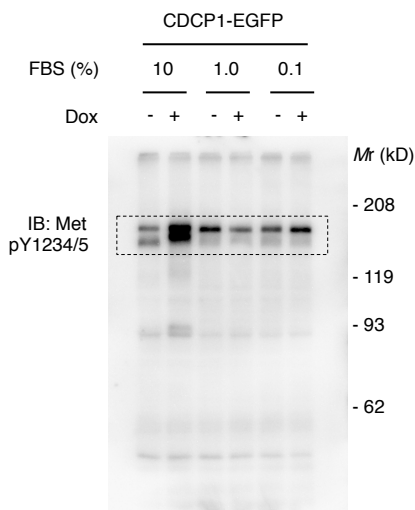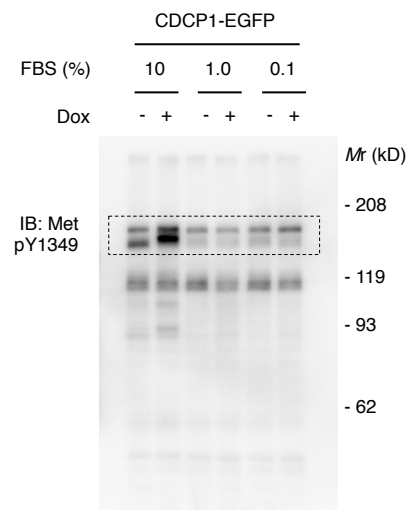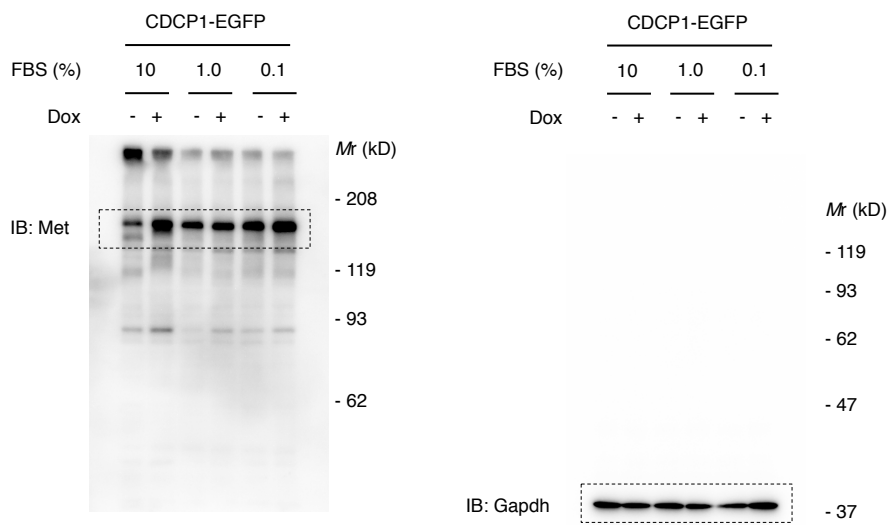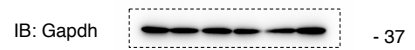

Supplement: Supplementary file 13 [file LSA-2020-00832_SdataFS8.pdf]

Figure S9D

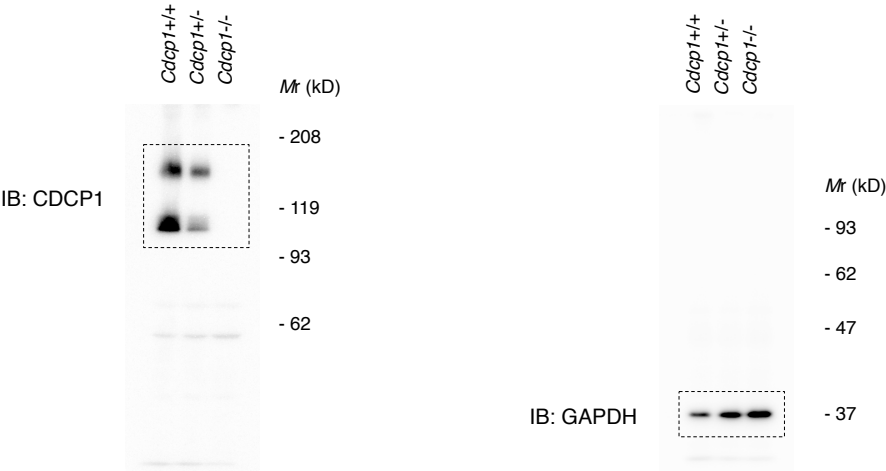

Supplement: Supplementary file 14 [file LSA-2020-00832_SdataFS9.pdf]
